# Medicines for treatment of older people in guidelines and essential medicines lists, WHO African Region

**DOI:** 10.2471/BLT.25.294289

**Published:** 2026-01-14

**Authors:** Ke Wei Foong, Amelia Paveley, Isabella Alcock, Pamela Gorejena-Chidawanyika, Celia L Gregson, Grace ME Pearson

**Affiliations:** a Older Persons Unit, Royal United Hospitals Bath NHS Foundation Trust, Bath, England.; b The Global Health and Ageing Research Unit, University of Bristol Medical School, Learning & Research Building, Southmead Hospital, Bristol, BS10 5FN, England.; c Faculty of Medicine and Health Sciences, University of Zimbabwe, Harare, Zimbabwe.

## Abstract

**Objective:**

To assess the geriatric medicine content in national standard treatment guidelines and essential medicines lists across the 47 Member States of the World Health Organization (WHO) African Region.

**Methods:**

Until 28 June 2025, we searched for national guidelines and lists in the Global Essential Medicines database and the WHO Repository of National Essential Medicines Lists. We examined each document for a geriatric medicine chapter and guidance on the management of frailty, falls, palliative care, osteoporosis, parkinsonism, incontinence, delirium, dementia and polypharmacy. We obtained country-level data from the World Bank and WHO. Using *χ^2^*-tests, we determined associations between country-level metrics and geriatric medicine content.

**Findings:**

We obtained a standard treatment guideline or essential medicines list from all 47 countries. Six (13%) documents contained a geriatric medicine chapter (five in English, one in French). Guidance on parkinsonism was the most common (42 documents; 89%), while guidance on frailty was the least common (three documents; 6%). Guidance on dementia was associated with current and predicted percentage population aged 65 years or older (*P*-value: 0.05), while guidance on palliative care was associated with healthy life expectancy both at birth and at age 60 years (*P*-value: 0.02 and *P*-value: 0.02, respectively).

**Conclusion:**

Countries in the African Region with a higher proportion of people older than 65 years were more likely to include geriatric medicine content in their standard treatment guidelines and essential medicines lists. There is considerable potential for expanding guidance on management of common geriatric conditions, such as frailty, incontinence and polypharmacy.

## Résumé

**Objectif:**

Évaluer les contenus liés à la gériatrie dans les directives thérapeutiques standard à l’échelle nationale et dans les listes de médicaments essentiels des 47 États membres de la région Afrique de l’Organisation mondiale de la Santé (OMS).

**Méthodes:**

Jusqu’au 28 juin 2025, nous avons recherché des directives et listes nationales dans la base de données mondiale sur les médicaments essentiels et le répertoire des listes nationales de médicaments essentiels de l’OMS. Nous avons examiné chaque document afin d’y trouver un chapitre consacré à la gériatrie et des recommandations sur la prise en charge de la fragilité, des chutes, des soins palliatifs, de l’ostéoporose, du syndrome parkinsonien, de l’incontinence, du délire, de la démence et de la polymédication. Nous avons obtenu des données par pays individuel auprès de la Banque mondiale et de l’OMS. Nous avons appliqué des tests du *χ^2^* pour déterminer les associations entre les indicateurs par pays et les contenus relatifs à la gériatrie.

**Résultats:**

Nous avons obtenu, pour les 47 pays, une directive thérapeutique standard ou une liste de médicaments essentiels. Six documents (13%) contenaient un chapitre sur la gériatrie (cinq en anglais, un en français). Les recommandations sur le syndrome parkinsonien étaient les plus courantes (42 documents, soit 89%), tandis que celles sur la fragilité étaient les moins courantes (trois documents, soit 6%). Les recommandations concernant la démence étaient associées au pourcentage actuel et prévu de la population âgée de 65 ans ou plus (valeur *P*: 0,05), tandis que celles sur les soins palliatifs étaient associées à une espérance de vie en bonne santé à la naissance et à l’âge de 60 ans (valeur *P*: 0,02 et valeur *P*: 0,02, respectivement).

**Conclusion:**

Les pays de la région Afrique ayant une proportion plus élevée de personnes âgées de plus de 65 ans étaient davantage susceptibles d’inclure des contenus relatifs à la gériatrie dans leurs directives thérapeutiques standard et leurs listes de médicaments essentiels. Il existe un potentiel considérable pour élargir les recommandations sur la prise en charge des affections gériatriques courantes, telles que la fragilité, l’incontinence et la polymédication.

## Resumen

**Objetivo:**

Evaluar el contenido de medicina geriátrica en las guías nacionales de tratamiento estándar y en las listas de medicamentos esenciales de los 47 Estados Miembros de la Región de África de la Organización Mundial de la Salud (OMS).

**Métodos:**

Hasta el 28 de junio de 2025, se buscaron guías nacionales y listas en la base de datos Global Essential Medicines y en el Repositorio de Listas Nacionales de Medicamentos Esenciales de la OMS. Se examinó cada documento para identificar la presencia de un capítulo de medicina geriátrica y orientaciones sobre el manejo de la fragilidad, las caídas, los cuidados paliativos, la osteoporosis, el parkinsonismo, la incontinencia, el delirium, la demencia y la polifarmacia. Se obtuvieron datos a nivel de país del Banco Mundial y de la OMS. Mediante pruebas de *χ^2^*, se determinaron las asociaciones entre los indicadores a nivel de país y el contenido de medicina geriátrica.

**Resultados:**

Se obtuvo una guía de tratamiento estándar o una lista de medicamentos esenciales de los 47 países. Seis documentos (13%) contenían un capítulo de medicina geriátrica (cinco en inglés y uno en francés). Las orientaciones sobre el parkinsonismo fueron las más frecuentes (42 documentos; 89%), mientras que las relativas a la fragilidad fueron las menos comunes (tres documentos; 6%). La inclusión de orientaciones sobre la demencia se asoció con el porcentaje actual y proyectado de la población de 65 años o más (valor de *p*: 0,05), mientras que las orientaciones sobre cuidados paliativos se asociaron con la esperanza de vida saludable tanto al nacer como a los 60 años (valor de *p*: 0,02 y valor de *p*: 0,02, respectivamente).

**Conclusión:**

Los países de la Región de África con un mayor porcentaje de personas mayores de 65 años tenían más probabilidades de incluir contenidos de medicina geriátrica en sus guías de tratamiento estándar y en sus listas de medicamentos esenciales. Existe un amplio potencial para ampliar las orientaciones sobre el manejo de afecciones geriátricas frecuentes, como la fragilidad, la incontinencia y la polifarmacia.

## ملخص

**الغرض:**

تقييم محتوى طب الشيخوخة في المبادئ التوجيهية الوطنية الموحدة للعلاج، وقوائم الأدوية الأساسية في الدول الأعضاء البالغ عددها 47 دولة في المنطقة الأفريقية التابعة لمنظمة الصحة العالمية (WHO).

**الطريقة:**

حتى 28 يونيو/حزيران 2025، قمنا بالبحث عن المبادئ التوجيهية والقوائم الوطنية في قاعدة بيانات الأدوية الأساسية العالمية ومستودع منظمة الصحة العالمية لقوائم الأدوية الأساسية الوطنية. قمنا بفحص كل مستند بحثًا عن فصل خاص بطب الشيخوخة، وإرشادات حول إدارة الوهن، والسقوط، والرعاية التلطيفية، وهشاشة العظام، ومرض باركنسون، وسلس البول، والهذيان، والخرف، وتعدد الأدوية. وحصلنا على بيانات على مستوى الدول من البنك الدولي ومنظمة الصحة العالمية. باستخدام اختبارات *χ ^2 ^*، قمنا بتحديد الارتباطات بين المقاييس على مستوى الدول، ومحتوى طب الشيخوخة.

**النتائج:**

حصلنا على مبدأ توجيهي للعلاج القياسي، أو قائمة أدوية أساسية من جميع الدول البالغ عددها 47 دولة. احتوت ست مستندات (بنسبة %13) على فصلٍ خاص بطب الشيخوخة (خمس منها باللغة الإنجليزية، وواحدة باللغة الفرنسية). وكان التوجيه المتعلق بمرض باركنسون هو الأكثر شيوعًا (42 مستندًا؛ %89)، بينما كان التوجيه المتعلق بالوهن هو الأقل شيوعًا (ثلاث مستندات؛ %6). وارتبط التوجيه المتعلق بالخرف بنسبة السكان الحالية والمتوقعة ممن تبلغ أعمارهم 65 عامًا فأكثر (قيمة *الاحتمال *: 0.05)، في حين ارتبط التوجيه المتعلق بالرعاية التلطيفية بمتوسط العمر المتوقع بصحة جيدة عند الولادة، وعند بلوغ سن 60 عامًا (قيمة *الاحتمال *: 0.02، وقيمة *الاحتمال *: 0.02، على التوالي).

**الاستنتاج:**

كانت الدول في المنطقة الأفريقية التي تضم نسبة أعلى من السكان الذين تزيد أعمارهم عن 65 عامًا، أكثر ميلًا لإدراج محتوى طب الشيخوخة في إرشادات العلاج القياسي، وقوائم الأدوية الأساسية. هناك إمكانات كبيرة لتوسيع نطاق التوجيه المتعلق بإدارة الحالات الشائعة لدى كبار السن، مثل الوهن وسلس البول وتعدد الأدوية.

## 摘要

**目的:**

旨在评估世卫组织 (WHO) 非洲区域 47 个会员国的国家标准治疗指南和基本药物清单中的老年医学内容。

**方法:**

截至 2025 年 6 月 28 日，我们搜索了全球基本药物数据库以及世卫组织国家基本药物清单储存库中的国家指南和药物清单。我们审阅了每份文件，以查找老年医学章节以及针对虚弱、跌倒、姑息治疗、骨质疏松症、帕金森综合征、失禁、谵妄、痴呆症和多重用药的管理指导意见。我们从世界银行和世卫组织处获取了各国的相关数据。通过采用 χ2 检验，我们确定了各国指标与老年医学内容之间的关联性。

**结果:**

我们已将 47 个国家的标准治疗指南或基本药物清单全部收集到位。只有六份（占 13%）文件包含了老年医学章节（五份为英文版，一份为法语版）。针对帕金森综合征的管理指导意见最为常见（42 份文件；占 89%），而针对虚弱的管理指导意见则最为罕见（三份文件；占 6%）。针对痴呆症的管理指导意见与当前及预测的年龄为 65 岁或以上的人口比例（P 值：0.05）挂钩，而针对姑息治疗的管理指导意见则与出生时以及年满 60 岁时的健康预期寿命（分别为 P 值：0.02 和 P 值：0.02）挂钩。

**结论:**

在非洲区域，年龄在 65 岁以上的人口所占比例较高的国家更有可能将老年医学内容并入其标准治疗指南和基本药物清单中。这些国家极有可能会增加针对虚弱、失禁和多重用药等常见老年疾病的管理指导意见**。**

## Резюме

**Цель:**

Оценить, какая информация, относящаяся к гериатрической медицине, включена в национальные стандартные руководства по лечению пациентов и перечни основных лекарственных средств в 47 государствах-членах Африканского региона Всемирной организации здравоохранения (ВОЗ).

**Методы:**

До 28 июня 2025 года авторы проводили поиск по национальным руководствам и перечням препаратов в Глобальной базе данных основных лекарственных средств и в Репозитории национальных перечней основных лекарственных средств ВОЗ. Каждый документ изучался на наличие раздела по гериатрической медицине и рекомендаций по ведению случаев астении, падений, остеопороза, паркинсонизма, недержания, делирия, деменции, по оказанию паллиативной помощи и проведению полипрагмазии. Данные на уровне отдельных стран были получены из Всемирного банка и ВОЗ. Используя критерий *χ^2^*, авторы определили связь между показателями на уровне страны и содержанием раздела по гериатрической медицине.

**Результаты:**

Стандартные руководства по лечению или перечни основных лекарственных средств были получены от всех 47 стран. Шесть документов (13%) содержали раздел, посвященный гериатрической медицине (пять на английском языке, один на французском). Наиболее часто встречались рекомендации по лечению паркинсонизма (42 документа, 89%), тогда как наиболее редко встречались рекомендации, касающиеся астении (три документа, 6%). Рекомендации относительно деменции ассоциировались с текущей и прогнозируемой долей населения в возрасте 65 лет или старше (*Р*-значение: 0,05), тогда как рекомендации относительно паллиативного ухода были связаны с ожидаемой продолжительностью здоровой жизни как в момент рождения, так и в возрасте 60 лет (*Р*-значение: 0,02 и *Р*-значение: 0,02 соответственно).

**Вывод:**

Страны Африканского региона с более высокой долей населения старше 65 лет с большей вероятностью включали информацию о гериатрической медицине в стандартные руководства по лечению пациентов и перечни основных лекарственных средств. Имеется значительный потенциал для расширения рекомендаций по ведению таких распространенных гериатрических состояний, как астения, недержание и потребность в полипрагмазии.

## Introduction

The United Nations projects that the number of adults older than 65 years in Africa will rise from 41 million in 2025 to 103 million by 2050, making African populations the fastest ageing in the world.^1^^–^^3^ Ageing, especially rapid ageing, poses a challenge to health and social care, as the coexistence of multiple long-term conditions (multimorbidity), the prescription of multiple medications (polypharmacy) and disability all increase with age. Together, these factors introduce complexity into the care of older people that requires multidisciplinary input with specialist knowledge and skills. In the initiative *Decade of healthy ageing 2021–2030*,^4^ the World Health Organization (WHO) has proposed a person-centred approach towards multidimensional assessment and management of the intrinsic capacities of older people within the environment in which they reside. This approach emphasizes the promotion and maintenance of health to prevent long-term loss of functional ability.^5^ Despite this initiative, care of older people remains a nascent field in Africa. More than a decade since the adoption of the African Union’s *AU policy framework and plan of action on ageing* in 2010, the care of older people remains variable and often deprioritized.^6^

Geriatric medicine is a branch of general medicine focusing on optimizing the health of older people through patient-centred, multidisciplinary comprehensive geriatric assessment.^7^ Geriatric teams provide specialist management for common geriatric conditions such as falls, frailty, bone health, movement disorders, delirium, dementia, incontinence, polypharmacy and end-of-life care.^8^ While the African framework and plan of action recognized a need for geriatric medicine to be included in health-care training, most African countries lack formal undergraduate and postgraduate training programmes, and many have no practising geriatricians.^9^^–^^11^

Country-level standard treatment guidelines and essential medicines lists, often developed based on the *WHO Model list of essential medicines*, are frequently used in all health-care settings across the African Region.^12^^,^^13^ These are intended as a guide to support national and regional authorities in choosing essential medicines that are effective, safe and cost-effective, and that reflect disease prevalence and clinical need. As such, they provide information about national health-care priorities. The minimum guidance is an essential medicines list: a list of recommended and available medications in the country. Increasingly, these documents have evolved into standard treatment guidelines, providing guidance on the diagnosis, investigation and management of conditions, often divided into chapters based on single organs or diseases. In our experience, where these detailed standard treatment guidelines exist, they are frequently employed as a reference text in clinical teaching and practice. The guidelines therefore offer a mechanism for upskilling entire workforces, regardless of specialty.

In the absence of specific content on geriatric medicine, siloed guidance often fails to support the care of older people, whose multimorbidity and complex needs require a more holistic, interdisciplinary approach. To map these gaps in guidance and identify areas for improvement, we reviewed the geriatric medicine content in standard treatment guidelines and essential medicines lists across the WHO African Region.

## Methods

We performed an ecological study of standard treatment guidelines and essential medicines lists published by health authorities in the 47 Member States of the WHO African Region. 

### Data sources and collection

Until 28 June 2025, we searched for standard treatment guidelines and essential medicines lists in two freely available and searchable online repositories: the Global Essential Medicines database and WHO’s Repository of National Essential Medicines Lists.^14^^,^^15^ Where multiple versions of a country’s standard treatment guideline and essential medicines list were available, we used the most recently published. Where both a standard treatment guideline and an essential medicines list were available as separate documents, we used standard treatment guidelines as they offered more detail. Identified guidelines and lists published in English or French were examined by a native English or French speaker, respectively. Documents in other languages were translated into English using ChatGPT (OpenAI, San Francisco, United States of America) and then analysed by a native English speaker.

### Country characteristics

Until 17 December 2024, we obtained country-level data for the 47 countries from the World Bank open database.^16^ These included population variables relevant to older people, including percentage of population aged 65 years or older and old-age dependency ratio (defined as the ratio of older dependants, aged older than 64 years, to the working-age population, aged 15–64 years, expressed as the proportion of older dependants per 100 working-age population). We also included economic variables such as the United Nations classification of least-developed countries and health expenditure as percentage of gross domestic product (GDP). We obtained data on healthy life expectancy from WHO’s Maternal, Newborn, Child and Adolescent Health and Ageing data portal on 13 October 2025.^17^ From these online databases, we used the latest available data, with reference year varying across countries. 

### Outcome data

One author examined each document to identify whether it contained a geriatric medicine chapter and/or guidance on the management of nine geriatric conditions: (i) frailty; (ii) falls; (iii) palliative care; (iv) osteoporosis; (v) parkinsonism; (vi) incontinence; (vii) delirium; (viii) dementia; and (ix) polypharmacy.

These nine geriatric conditions reflect topics most commonly covered in educational content on ageing and geriatrics.^18^ We defined management as any content on diagnosis, investigation, and/or treatment for the aforementioned geriatric conditions, therefore management did not require an entire, dedicated chapter. In some cases, the available guidance was only a list of medications that can be used to treat the condition, for example, antiparkinsonian medicines listed in an essential medicines list, and was not tailored specifically to the care of older people. However, such guidance was still considered relevant if the prespecified geriatric condition was explicitly mentioned and if it met the definition of management above. Examples of excluded content, as not specific to the care of older people, include guidance on delirium tremens; acquired immunodeficiency syndrome (AIDS) dementia complex; and falls, where guidance frequently addressed injuries by any mechanism. Furthermore, we did not include a mentioned condition without any guidance provided on its management. For example, where incontinence was listed as a symptom of a seizure, or where osteoporosis was listed as a side effect of an antiretroviral drug. These parameters were prespecified with all authors before data extraction.

### Statistical analyses

We determined associations between country-level metrics and the geriatric medicine content in each country’s standard treatment guideline or essential medicines list, by dividing countries into reference and comparison groups based on the median value of each continuous metric. We used *χ^2^*-tests to compare the outcomes in each group. Following current best practices,^19^ we interpreted smaller *P*-values as indicating stronger evidence against the null hypothesis, rather than applying a fixed *P*-value threshold.

## Results

**Fig. 1:**
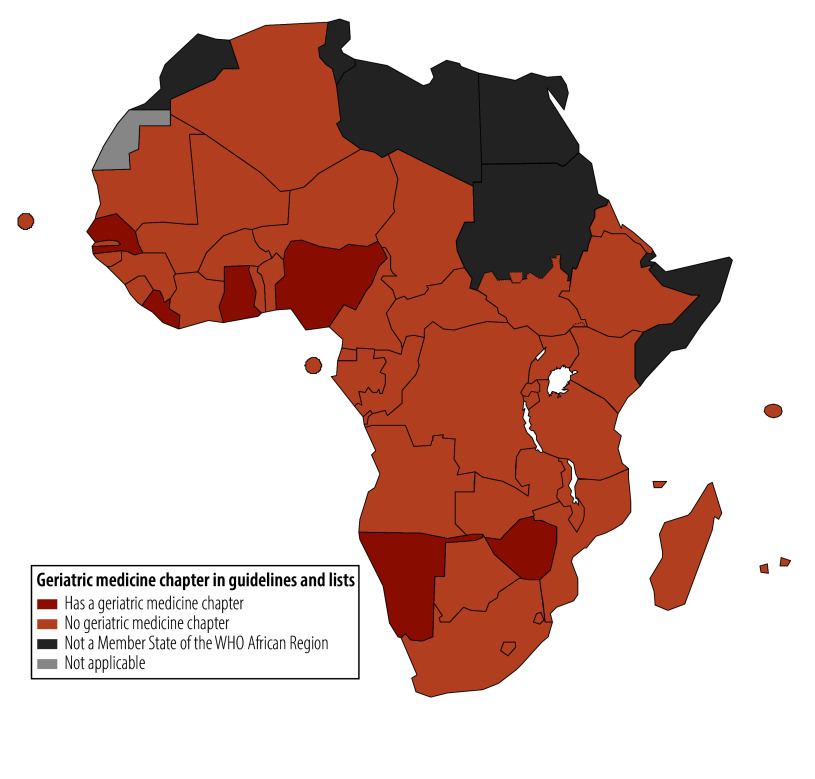
Availability of a chapter on geriatric medicine in either standard treatment guidelines or essential medicine lists, WHO African Region, 28 June 2025

We obtained standard treatment guidelines or essential medicines lists for all 47 Member States of the WHO African Region.^20^^–^^66^ Of these, 22 (47%) documents were published in English,^23^^,^^35^^–^^37^^,^^39^^,^^42^^–^^44^^,^^46^^,^^49^^,^^51^^,^^53^^,^^54^^,^^57^^–^^61^^,^^63^^–^^66^ 19 (40%) in French,^20^^,^^22^^,^^24^^,^^25^^,^^27^^–^^33^^,^^38^^,^^40^^,^^45^^,^^47^^,^^48^^,^^52^^,^^56^^,^^62^ five (11%) in Portuguese^21^^,^^26^^,^^41^^,^^50^^,^^55^ and one (2%) in Spanish.^34^ Only six (13%) documents contained a geriatric medicine chapter, which was more likely to be published in English (five documents in English;^39^^,^^44^^,^^51^^,^^53^^,^^66^ one document in French;^56^ *P*-value: 0.06; Fig. 1). Table 1 summarizes the associations between country-level metrics and the presence of a geriatric medicine chapter. An old-age dependency ratio of 5.6% or higher, a current population aged 65 years or older of 3.2% or higher, and a predicted population aged 65 years or older of 3.4% or higher tended to be associated with the presence of a geriatric medicine chapter, but statistical testing did not rule out the possibility that this association was due to chance. 

WHO: World Health Organization.

**Table 1:** Association between country-level metrics and presence of geriatric medicine chapter

Variable	Geriatric medicine chapter		*P*
	No	Yes	
**UN country classification**			
Classified as least developed	28	2	0.10
Not classified as least developed	13	4	
**Health expenditure as % of GDP^a^**			
< 4.5%	21	4	0.48
≥ 4.5%	20	2	
**Old-age dependency ratio^b^**			
< 5.6%	20	1	0.14
≥ 5.6%	21	5	
**% of population ≥ 65 years in 2023**			
< 3.2%	22	1	0.09
≥ 3.2%	19	5	
**% of predicted population ≥ 65 years in 2028**			
< 3.4%	20	1	0.14
≥ 3.4%	21	5	
**Healthy life expectancy at birth^c^**			
< 54.8 years	20	3	0.96
≥ 54.8 years	21	3	
**Healthy life expectancy at 60 years^d^**			
< 12.3 years	20	2	0.48
≥ 12.3 years	21	4	
**Recency of chapter update**			
Pre-2020	16	4	0.20
2020 onwards	25	2	
**Language **			
English	17	5	0.06
French, Portuguese or Spanish	24	1	

GDP: gross domestic product; UN: United Nations.

^a^ Current level of health expenditure, expressed as percentage of GDP.

^b^ Old-age dependency ratio is defined as the ratio of older dependants (aged > 64 years) to the working-age population (aged 15–64 years), expressed as the proportion of older dependants per 100 working-age population.

^c^ Average number of years in full health a person can expect to live from birth, in the year 2021.

^d^ Average number of years in full health a person can expect to live from 60 years of age based on current rates of ill-health and mortality, in the year 2021.

Notes: We used the United Nation’s classification of least-developed countries. Exposure variables are divided into reference and comparison groups based on the median value of each continuous metric. We obtained *P*-values through *χ^2^* tests.

**Fig. 2:**
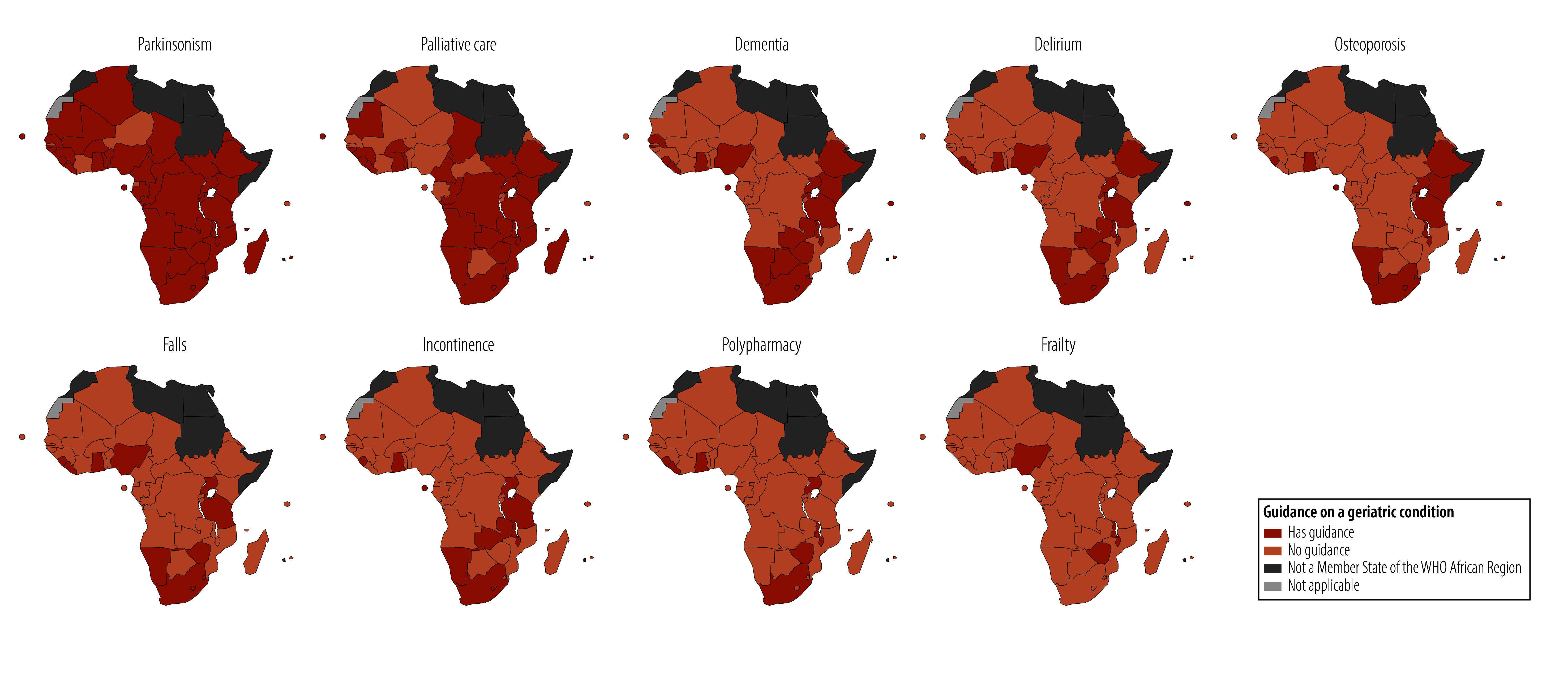
Availability of guidance on nine geriatric conditions, WHO African Region, 28 June 2025

Guidance on parkinsonism was found in most documents (42; 89%),^20^^–^^31^^,^^33^^,^^35^^–^^51^^,^^53^^–^^56^^,^^58^^–^^60^^,^^62^^–^^66^ while guidance on frailty was the least common (three documents; 6%).^46^^,^^53^^,^^66^ Table 2 summarizes the distribution of specialty chapters in which guidance on the various geriatric conditions was found; neurology and psychiatry showed the greatest overlap with geriatrics. Fig. 2 illustrates the countries that had a standard treatment guideline or essential medicines list containing guidance on each of the nine geriatric conditions. 

**Table 2:** Standard treatment guideline or essential medicines list chapters containing guidance on geriatric conditions, WHO African Region

Specialty chapter	No. of countries having guidance on the condition								
	Parkinsonism	Palliative care	Dementia	Delirium	Osteoporosis	Falls	Incontinence	Polypharmacy	Frailty
Geriatrics	1	–	3	1	1	2	1	3	2
Neurology	37	–	6	3	–	4	2	–	–
Psychiatry	3	–	11	11	–	1	–	1	1
Palliative care	–	20	–	–	–	–	1	–	–
Renal and urology	–	–	–	–	–	–	6	–	–
Oncology	–	8	–	–	–	–	–	–	–
Trauma	–	–	–	–	2	3	–	–	–
Musculoskeletal	–	–	–	–	3	–	–	–	–
Gynaecology	–	–	–	–	3	–	–	–	–
Prescribing	–	–	–	–	–	–	–	3	–
Endocrinology	–	–	–	–	4	–	–	–	–
Hepatology	–	1	–	–	–	–	–	–	–
Ophthalmology	1	–	–	–	–	–	–	–	–
Cardiovascular	–	–	–	–	–	1	–	–	–
Emergencies	–	–	–	1	–	–	–	–	–
Infectious diseases	–	–	1	–	–	–	–	–	–
**Total (%)**	**42 (89)**	**29 (62)**	**21 (45)**	**16 (34)**	**13 (28)**	**11 (23)**	**10 (21)**	**7 (15)**	**3 (6)**

WHO: World Health Organization.

WHO: World Health Organization.

Table 3 outlines the associations between country-level metrics and the presence of guidance on the pre-specified geriatric conditions. A current percentage population aged 65 years or older of 3.2% or more was associated with having guidance on dementia (14 documents; 30% versus seven documents; 15%; *P*-value: 0.05) as was a predicted population aged 65 years or older of 3.4% or more (15 documents; 32% versus six documents; 13%; *P*-value: 0.05). Similarly, guidance on palliative care was more likely in documents from countries where the healthy life expectancy at birth was less than 54.8 years (18 documents; 38% versus 11 documents; 23%; *P*-value: 0.02) and healthy life expectancy at age 60 years was less than 12.3 years (17 documents; 36% versus 12 documents; 26%; *P*-value: 0.04). 

**Table 3:** Associations of mentioning of geriatric conditions in guidelines and essential medicines lists with population ageing, publication timing and language, WHO African Region

Characteristic	Frailty				Falls				Palliative care				Osteoporosis				Parkinsonism				Incontinence				Delirium				Dementia				Polypharmacy		
	No	Yes	*P*		No	Yes	*P*		No	Yes	*P*		No	Yes	*P*		No	Yes	*P*		No	Yes	*P*		No	Yes	*P*		No	Yes	*P*		No	Yes	*P*
**UN country classification**																																			
Classified as least developed	29	1	0.26		25	5	0.15		11	19	0.76		23	7	0.38		2	28	0.24		23	7	0.65		21	9	0.44		19	11	0.14		26	4	0.69
Not classified as least developed	15	2			11	6			7	10			11	6			3	14			14	3			10	7			7	10			14	3	
**Health expenditure as % of GDP**																																			
< 4.5%	23	2	0.63		20	5	0.56		12	13	0.15		20	5	0.21		5	20	0.03		22	3	0.10		18	7	0.35		16	9	0.20		22	3	0.55
≥ 4.5%	21	1			16	6			6	16			14	8			0	22			15	7			13	9			10	12			18	4	
**Old-age dependency ratio^a^**																																			
< 5.6	19	2	0.43		18	3	0.19		7	14	0.53		16	5	0.60		3	18	0.47		17	4	0.74		15	6	0.48		14	7	0.16		18	3	0.92
≥ 5.6	25	1			18	8			11	15			18	8			2	24			20	6			16	10			12	14			22	4	
**% of population ≥ 65 years in 2023**																																			
< 3.2%	21	2	0.53		19	4	0.34		8	15	0.63		18	5	0.37		3	20	0.60		18	5	0.94		17	6	0.26		16	7	0.05		20	3	0.73
≥ 3.2%	23	1			17	7			10	14			16	8			2	22			19	5			14	10			10	14			20	4	
**% of predicted population ≥ 65 years in 2028**																																			
< 3.4%	19	2	0.43		18	3	0.27		7	14	0.53		17	4	0.24		2	19	0.82		17	4	0.74		16	5	0.18		15	6	0.05		19	2	0.35
≥ 3.4%	25	1			18	8			11	15			17	9			3	23			20	6			15	11			11	15			21	5	
**Healthy life expectancy at birth**																																			
< 54.8 years	21	2	0.53		16	7	0.27		5	18	0.02		17	6	0.81		2	21	0.67		17	6	0.43		14	9	0.47		13	10	0.87		18	5	0.20
≥ 54.8 years	23	1			20	4			13	11			17	7			3	21			20	4			17	7			13	11			22	2	
**Healthy life expectancy at 60 years**																																			
< 12.3 years	20	2	0.48		17	5	0.92		5	17	0.04		16	6	0.96		1	21	0.20		17	5	0.82		15	7	0.76		13	9	0.63		19	3	0.82
≥ 12.3 years	24	1			19	6			13	12			18	7			4	21			20	5			16	9			13	12			21	4	
**Recency of update**																																			
Pre-2020	19	1	0.74		14	6	0.36		9	11	0.42		16	4	0.31		4	16	0.07		16	4	0.85		12	8	0.46		11	9	0.97		17	3	0.99
2020 onwards	25	2			22	5			9	18			18	9			1	26			21	6			19	8			15	12			23	4	
**Language**																																			
English	19	3	0.06		11	11	< 0.01		6	16	0.15		10	12	< 0.01		2	20	0.75		13	9	< 0.01		6	16	< 0.01		3	19	< 0.01		15	7	< 0.01
French, Portuguese, Spanish	25	0			25	0			12	13			24	1			3	22			24	1			25	0			23	2			25	0	

GDP: gross domestic product; UN: United Nations; WHO: World Health Organization.

^a^ Old-age dependency ratio is defined as the ratio of older dependants (aged > 64 years) to the working-age population (aged 15–64 years), expressed as the proportion of older dependants per 100 working-age population.

Standard treatment guideline and essential medicines lists published in or after the year 2020 were more likely to contain guidance on parkinsonism (26 documents; 55% versus 16 documents; 34%; *P*-value: 0.07; Table 3). Only five documents lacked guidance on parkinsonism, all from countries where health expenditure as a percentage of GDP was < 4.5% (*P*-value: 0.03; Table 3). Publication in a language other than English was associated with the absence of guidance on multiple geriatric conditions (Table 3). Guidance available only in English-language documents included frailty (three documents), polypharmacy (seven documents), falls (11 documents) and delirium (16 documents). Guidance available almost exclusively in English-language documents included dementia (19 in English, one in French and one in Portuguese), osteoporosis (12 in English and one in Portuguese) and incontinence (nine in English and one in Portuguese).

## Discussion

We found that only six of 47 analysed standard treatment guidelines and essential medicines lists had a chapter on geriatric medicine, and that the presence of such a chapter tended to occur in countries with a higher proportion of older people in their populations, although an association due to chance could not excluded. Similarly, the presence of guidance on dementia was associated with the proportions of current and predicted population of people aged 65 years or older, and the presence of guidance on palliative care was associated with healthy life expectancy. These findings suggest that health authorities recognize the challenges posed by ageing populations and have adapted some national strategies and priorities to address them.^4^^,^^67^ Similarly, the association between the presence of guidance on palliative and dementia care and country-level metrics likely reflects the proportionately greater burden or, more likely, greater recognition of these age-related conditions and associated specialist health-care needs. In contrast, conditions such as frailty, incontinence and polypharmacy were rarely represented. When developing future standard treatment guidelines and essential medicines lists, lessons learnt from palliative and dementia care should be considered to improve awareness and knowledge of other important geriatric conditions. The recently published WHO framework for continence assessment may also help raise awareness of this need.^5^

For the 42 countries that did not have a chapter on geriatric medicine in their standard treatment guideline or essential medicines list, we hypothesize that the reason for exclusion is multifactorial: (i) many countries lack geriatrics expertise;^9^^–^^11^ (ii) the perceived low prestige of geriatric medicine may lead to conscious or unconscious exclusion of geriatricians from the development of these documents;^68^^–^^70^ (iii) geriatric care is often deprioritized in settings where constrained resources must be allocated across multiple competing demands;^71^ and (iv) ageism is widespread, making it acceptable to overlook the needs of older people.^72^

While we were reassured to find that all geriatric conditions were covered in English-language documents, this coverage occurred to varying extents. Guidance on parkinsonism was the most common, while guidance on frailty was the least covered. This difference likely reflects the fact that neurology is a more widely established discipline than geriatric medicine in African countries. The finding also reflects the greater level of funding available for Parkinson care, as we found that the only countries without guidance on parkinsonism in their standard treatment guidelines or essential medicines lists had health expenditures of less than 4.5% of GDP. Although we identified geriatric content, some guidance was not appropriate or tailored to the care of older people. For example, pharmacological sedation for delirium was recommended under neurology, despite this practice not being the recommended standard of care for more than 20 years.^73^ Parkinsonian disorders remain the remit of neurologists in the low- and middle-income countries, while in high-income countries, care models have moved towards shared care between neurology and geriatrics. Geriatricians therefore have the opportunity to take ownership of existing geriatric content by developing a dedicated chapter or by collaborating to improve content within existing chapters. However, due diligence is needed to ensure that country-level guidance is implementable within existing health-care contexts, with consideration given to the availability of resources and workforce structures. For example, in many countries, nurses provide front-line health care to older people and need to be enabled to prescribe medication in this role.

Moreover, we found an underrepresentation of osteoporosis and polypharmacy, despite both being associated with human immunodeficiency virus (HIV) infection. This finding demonstrates a disconnect between management of a highly prevalent communicable disease and its longer-term sequelae. This mismatch may worsen in the current climate of defunding HIV programmes.^74^ One way to improve care could be to incorporate guidance on these topics into existing HIV chapters. Another notable finding was the association between the presence of parkinsonism guidance and publication or update after 2020. This association may be explained by the coronavirus disease 2019 (COVID-19) pandemic, which changed health-care practice worldwide. This association may also be linked to the publication of the WHO report *Parkinson disease: a public health approach* in 2022.^75^^,^^76^ Again, lessons can be learnt from the rapid adaptations in health-care delivery to a pandemic, which can inform efforts to improve health-care services and guidance in geriatric medicine.

Frailty is a core clinical condition in geriatric medicine. For many years, however, a consensus definition was lacking. The condition is now generally understood as the age-related accumulation of deficits in physiological reserve and function, which confers increased vulnerability to adverse health outcomes when exposed to relatively minor stressors.^77^ The prevalence of frailty in people older than 50 years in Africa has been estimated to be 5–60%, showing a high level of health- and social-care need, despite difficulties in detecting frailty.^78^^,^^79^ Older people living with frailty present with atypical and nonspecific signs and symptoms of disease, which complicates diagnosis, investigation and management.^77^ Frailty has been associated with substantial morbidity and mortality,^77^^,^^79^ highlighting the importance of correct diagnosis and management. This challenge includes the sharing of specialist expertise in standard treatment guidelines and essential medicines lists.

To improve care for older people in the African Region, we must improve the transfer of knowledge from regions with a high level of geriatrics expertise and experience of geriatric care to health authorities and health workers in the African Region. Indeed, education and training, two of the priority areas of the *AU policy framework and plan of action on ageing*,^6^ can be achieved through collaboration and equitable partnerships in the development of standard treatment guidelines and essential medicines lists. For instance, when recently updating the standard treatment guideline in Zimbabwe, geriatricians trained in high-income countries collaborated with general physicians trained in low- and middle-income countries to improve and expand the chapter on geriatric medicine in a clinically and culturally relevant manner (National Medicine and Therapeutics Policy Advisory Committee, Ministry of Health and Child Care, Zimbabwe, unpublished report, November 2025). To improve the care of older people, standard treatment guidelines must be complemented with undergraduate and postgraduate training of health workers, improved access to health-care facilities and medicines, proactive clinical assessments (for example, as proposed by WHO)^5^ and implementation of long-term care models.

This study has several limitations. First, we did not examine content for visual and hearing impairments, malnutrition and depression. These are areas identified by WHO as core to the comprehensive assessment of older people, but for the purposes of our study, were too broad to include.^5^ Second, we only mapped the presence of written material and did not analyse the quality and accuracy of the content. Our focus was on medicines and did not consider guidance on access to assistive technologies which are also important to support independence and well-being of ageing populations.

In conclusion, there is a mismatch between global population ageing and capacity of health workforces to adequately and appropriately care for older people with complex needs. One way of redressing this imbalance and upskilling health workers is by promoting geriatric medicine content in standard treatment guidelines and essential medicines lists. We found that countries in the WHO African Region with a higher proportion of older people were more likely to include geriatric medicine content in their standard treatment guidelines or essential medicines lists. Including such content in more guidelines and lists would be an appropriate response to demographic shifts, as populations in the region continue to age. However, only a minority of the examined guidelines and lists contained a dedicated chapter on geriatric medicine, and guidance on geriatric conditions was otherwise distributed across traditionally recognized specialities. Therefore, there remains considerable potential to expand guidance on the management of key geriatric conditions, such as frailty, incontinence and polypharmacy, either by including them into existing specialty chapters or by introducing new chapters on geriatric medicine.

## References

[1] Ageing in Africa. Lancet Healthy Longev. 2025 Sep;6(9):100779. 10.1016/j.lanhl.2025.100779 41101322

[2] The Lancet Diabetes Endocrinology. The ageing of Africa. Lancet Diabetes Endocrinol. 2016 Jan;4(1):1. 10.1016/S2213-8587(15)00484-2 26681188

[3] 2024 revision of world population prospects. New York: United Nations; 2025. Available from: https://population.un.org/wpp/ [cited 2025 Nov 25].

[4] WHO's work on the UN Decade of Healthy Ageing (2021-2030). Geneva: World Health Organization; 2025. Available from: https://www.who.int/initiatives/decade-of-healthy-ageing [cited 2025 Nov 20].

[5] Integrated care for older people approach (ICOPE). Geneva: World Health Organization; 2024. Available from: https://www.who.int/teams/maternal-newborn-child-adolescent-health-and-ageing/ageing-and-health/integrated-care-for-older-people-icope [cited 2025 Oct 22].

[6] AU Policy Framework and Plan of Action on Ageing. London: HelpAge International; 2010. Available from: https://www.helpage.org/resource/au-policy-framework-and-plan-of-action-on-ageing/ [cited 2025 Apr 1].

[7] Cesari M, Amuthavalli Thiyagarajan J, Cherubini A, Angel Acanfora M, Assantachai P, Barbagallo M, et al. Defining the role and reach of a geriatrician. Lancet Healthy Longev. 2024 5(11)100644. 10.1016/j.lanhl.2024.100644 39374610 PMC11602445

[8] Nickel C, Arendts G, Lucke J, Mooijaart S. Geriatric syndromes. In: Conroy S, Carpenter C, Banerjee J, editors. Silver Book II. London: British Geriatrics Society; 2021. Available from: https://www.bgs.org.uk/resources/silver-book-ii-geriatric-syndromes [cited 2025 Apr 1].

[9] Dotchin CL, Akinyemi RO, Gray WK, Walker RW. Geriatric medicine: services and training in Africa. Age Ageing. 2013 Jan;42(1):124–8. 10.1093/ageing/afs119 23027519

[10] Frost L, Liddie Navarro A, Lynch M, Campbell M, Orcutt M, Trelfa A, et al. Care of the elderly: survey of teaching in an aging sub-Saharan Africa. Gerontol Geriatr Educ. 2015;36(1):14–29. 10.1080/02701960.2014.925886 24884474

[11] Pearson GME, Ben-Shlomo Y, Henderson EJ. A narrative overview of undergraduate geriatric medicine education worldwide. Eur Geriatr Med. 2024 Oct;15(5):1533–40. 10.1007/s41999-024-01055-1 39317883 PMC11614947

[12] WHO Model List of Essential Medicines - 23rd List, 2023. Geneva: World Health Organization; 2023. Available from: https://www.who.int/publications/i/item/WHO-MHP-HPS-EML-2023.02 [cited 2025 Apr 1].

[13] Model list of essential medicines [internet]. Geneva: World Health Organization; 2025. Available from: https://list.essentialmeds.org/ [cited 2025 Apr 1].

[14] Global essential medicines [internet]. Geneva: World Health Organization; 2025. Available from: https://global.essentialmeds.org/dashboard/countries [cited 2025 Apr 1].

[15] Repository of national essential medicines lists (nEMLs) [internet]. Geneva: World Health Organization; 2024. Available from: https://www.who.int/teams/health-product-policy-and-standards/assistive-and-medical-technology/essential-medicines/national-emls [cited 2025 Jul 7].

[16] World Bank open data [internet]. Washington, DC: World Bank Group; 2025. Available from: https://data.worldbank.org/ [cited 2025 Apr 1].

[17] Maternal, newborn, child and adolescent health and ageing. Geneva: World Health Organization; 2025. Available from: https://platform.who.int/data/maternal-newborn-child-adolescent-ageing/indicator-explorer-new/mca/ [cited 2025 Oct 22].

[18] Masud T, Blundell A, Gordon AL, Mulpeter K, Roller R, Singler K, et al. European undergraduate curriculum in geriatric medicine developed using an international modified Delphi technique. Age Ageing. 2014 Sep;43(5):695–702. 10.1093/ageing/afu019 24603283 PMC4143490

[19] Sterne JAC, Davey Smith G. Sifting the evidence-what’s wrong with significance tests? BMJ. 2001 Jan 27;322(7280):226–31. 10.1136/bmj.322.7280.226 11159626 PMC1119478

[20] Algeria: liste des médicaments remboursables par la sécurité sociale 2023 (French). Algiers: Ministry of Labour, Employment and Social Security; 2023. Available from: https://www.who.int/publications/m/item/algeria--la-liste-des-m-dicaments-remboursables-par-la-s-curit--sociale-2023-(french) [cited 2025 Oct 15].

[21] Angola: lista nacional de medicamentos essenciais 2021 (Portuguese). Luanda: Ministry of Health, Republic of Angola; 2021. Available from: https://www.who.int/publications/m/item/angola--lista-nacional-de-medicamentos-essenciais-2021-(portugues) [cited 2025 Oct 15].

[22] Benin: Liste Nationale des Médicaments Essentiels Enfants et Adultes 2018 (French). Cotonou: Ministry of Health, Republic of Benin; 2018. Available from: https://www.who.int/publications/m/item/benin--liste-nationale-des-medicaments-essentiels-enfants-et-adultes-2018-(french) [cited 2025 Oct 15].

[23] Botswana: essential medicines list (BEML) 2016 (English). 3rd edition electronic version. Gaborone: Ministry of Health Botswana; 2016. Available from: https://www.who.int/publications/m/item/botswana--botswana-essential-medicines-list-(beml)-2016-(english) [cited 2025 Oct 15].

[24] Burkina Faso: liste nationale des médicaments essentiels et autres produits de santé 2023 (French). Ouagadougou: Ministry of Health and Public Hygiene; 2023. Available from: https://www.who.int/publications/m/item/burkina-faso--liste-nationale-des-medicamentsessentiels-et-autres-produits-de-sante-2023-(french) [cited 2025 Oct 15].

[25] Burundi: liste nationale des médicaments essentiels 2022 (French). Bujumbura: Ministry of Public Health and the Fight Against AIDS; 2022. Available from: https://www.who.int/publications/m/item/burundi--liste-nationale-des-medicaments-essentiels-au-burundi-2022-(french) [cited 2025 Oct 15].

[26] Cabo Verde: lista nacional de medicamentos essenciais 2018 (Portuguese). Praia: Republic of Cabo Verde; 2018. Available from: https://www.who.int/publications/m/item/capo-verde--lista-nacional-de-medicamentos-essenciais-2018-(portugues) [cited 2025 Oct 15].

[27] Cameroon: liste nationale des médicaments et autres produits pharmaceutiques essentiels 2022 (French). Yaoundé: Ministry of Public Health, Republic of Cameroon; 2022. Available from: https://www.who.int/publications/m/item/cameroon--liste-nationale-des-medicaments-et-autres-produits-pharmaceutiques-essentiels-2017-(french) [cited 2025 Oct 15].

[28] Central African Republic: liste nationale des médicaments essentiels et dispositifs medicaux de la RCA 2017 (French). Bangui: Central African Republic; 2017. Available from: https://www.who.int/publications/m/item/central-african-republic--liste-nationale-des-medicaments-essentiels-et-dispositifs-medicaux-de-la-rca-2017-(french) [cited 2025 Oct 15].

[29] Chad: Liste Nationale des Médicaments Essentiels et Autres Produits de Santé 2022 (French). N'Djamena: Ministry of Public Health and National Solidarity; 2022. Available from: https://www.who.int/publications/m/item/chad--liste-nationale-des-medicaments-essentiels-et-autres-produits-de-sante-2020-(french) [cited 2025 Oct 15].

[30] Comoros: liste nationale des médicaments essentiels adulte et pédiatrique 2020 (French). Moroni: Ministry of Health, Solidarity, social protection and gender promotion, Union of the Comoros; 2022. Available from: https://www.who.int/publications/m/item/comoros--liste-nationale-des-medicaments-essentiels-adulte-et-pediatrique-2014-(french) [cited 2025 Oct 15].

[31] Congo: liste nationale des médicaments essentiels 2016 (French). Brazzaville: Ministry of Health and Population, Republic of Congo; 2016. Available from: https://www.who.int/publications/m/item/congo--liste-nationale-des-medicaments-essentiels-2016-(french) [cited 2025 Oct 15].

[32] Côte D'Ivoire: liste nationale des médicaments essentiels et du matériel biomédical (LNME) 2024 (French). Abidjan: Ministry of Health, Public hygiene and universal health coverage, Republic of Côte d'Ivoire; 2024. Available from: https://www.who.int/publications/m/item/cote-d-ivoire--liste-des-m-dicaments-pris-en-charge-par-la-cmu-(french) [cited 2025 Oct 15].

[33] Democratic Republic of Congo: Liste nationale des médicaments essentiels 2020 (French). Kinshasa: Ministry of Health, Democratic Republic of Congo; 2020. Available from: https://www.who.int/publications/m/item/democratic-republic-of-congo--liste-nationale-des-m-dicaments-essentiels-2020-(french) [cited 2025 Oct 15].

[34] Equatorial Guinea: lista nacional de medicamentos esenciales de Guinea Ecuatorial 2012 (Spanish). Malabo: Republic of Equatorial Guinea; 2012. Available from: https://www.who.int/publications/m/item/equatorial-guinea--lista-nacional-de-medicamentos-esenciales-de-guinea-ecuatorial-2012-(spanish) [cited 2025 Oct 15].

[35] Eritrea: national list of medicines 2010 (English). Asmara: Ministry of Health; 2010. Available from: https://www.who.int/publications/m/item/eritrea--eritrean-national-list-of-medicines-2010-(english) [cited 2025 Oct 15].

[36] Government of the Kingdom of Eswatini Ministry of Health, US President’s Emergency Plan for AIDS Relief (PEPFAR), USAID, Strengthening Pharmaceutical Systems (SPS) Program. Standard treatment guidelines and essential medicines list of common medical conditions in the Kingdom of Eswatini. Manzini: Ministry of Health; 2012. Available from: https://www.medbox.org/document/standard-treatment-guidelines-and-essential-medicines-list-of-common-medical-conditions-in-the-kingdom-of-swaziland [cited 2025 Oct 15].

[37] Standard treatment guidelines for general hospitals, 4th edition. Addis Ababa: Ministry of Health, Ethiopia: 2021. Available from: https://www.slideshare.net/slideshow/stg-2021pdf/252255886?from_action=download&slideshow_id=252255886 [cited 2025 Oct 15].

[38] Gabon: liste nationale des médicaments et dispositifs médicaux essentiels 2024 (French). Libreville: National Agency for Medicines and Other Health Products; 2024. Available from: https://www.who.int/publications/m/item/gabon--liste-nationale-des-medicaments-et-dispositifs-medicaux-essentiels-2019-(french) [cited 2025 Oct 15].

[39] Standard treatment guidelines, 7th edition. Accra: Ministry of Health Ghana National Drugs Program; 2017. Available from: https://www.moh.gov.gh/wp-content/uploads/2020/07/GHANA-STG-2017-1.pdf [cited 2025 Oct 15].

[40] Guinea: liste nationale des médicaments essentiels 2021 (French). Conakry: Ministry of Health; 2021. Available from: https://www.who.int/publications/m/item/guinea--liste-nationale-des-medicaments-essentiels-2021-(french) [cited 2025 Oct 15].

[41] Guinea-Bissau: lista de medicamentose produtos essenciais de saúde 2024 (Portuguese). Codex-Bissau: Ministry of Public Health, Government of Guinea-Bissau; 2024. Available from: https://www.who.int/publications/m/item/guinea-bissau--lista-nacional-de-medicamentos-essenciais-2020-(portugues) [cited 2025 Oct 15].

[42] Kenya: Essential Medicines List 2023 (English). Nairobi: Ministry of Health, Republic of Kenya; 2023. Available from: https://www.who.int/publications/m/item/kenya--essential-medicines-list-2023-(english) [cited 2025 Oct 15].

[43] Standard treatment guidelines for Lesotho, 3rd edition. Maseru: Ministry of Health, Lesotho; 2022. Available from: https://static1.squarespace.com/static/60bdea12fbad083dee4ffa2c/t/651fb589cf17c5449fc9ac66/1696576912929/STG+LESOTHO+Feb+2023+%281%29.pdf [cited 2025 Oct 15].

[44] National standard therapeutic guidelines and essential medicines list Liberia 2017, 2nd ed. Monrovia: Republic of Liberia, Ministry for Health and Social Welfare; 2017. Available from: https://www.medbox.org/pdf/5e148832db60a2044c2d4cf5 [cited 2025 Oct 15].

[45] Madagascar: médicaments essentiels et intrants de santé 2019 (French). LNMEIS 6ème edition. Antananarivo: Ministry of Public Health, Republic of Madagascar; 2019. Available from: https://www.who.int/publications/m/item/madagascar--m-dicaments-essentiels-et-intrants-de-sant--2019-(french) [cited 2025 Oct 15].

[46] Malawi standard treatment guidelines (MSTG), sixth edition. Lilongwe: Ministry of Health, Malawi; 2023. Available from: https://www.differentiatedservicedelivery.org/wp-content/uploads/MSTG-6th-Edition-2023-Final-Draft-CC-gn-2-edditi_230719_133059.pdf [cited 2025 Oct 15].

[47] Mali: liste nationale des médicaments essentiels 2024 (French). Bamako: Ministry of Health and Social Affairs, Republic of Mali; 2024. Available from: https://www.who.int/publications/m/item/mali--liste-nationale-des-m-dicaments-essentiels-par-niveau-mali-2019-(-french) [cited 2025 Oct 15].

[48] Mauritania: liste nationale des médicament essentiels 2024 (French). Nouakchott: Ministry of Health, Islamic Republic of Mauritania; 2024. Available from: https://www.who.int/publications/m/item/mauritania--liste-nationale-des-m-dicament-essentiels-par-niveau-de-la-pyramide-sanitaire-2021-(french) [cited 2025 Oct 15].

[49] Mauritius: Approved Drug List for Public Hospitals 2022 (English). LNMEIS 6ème édition. Port Louis: Government of Mauritius; 2022. Available from: https://www.who.int/publications/m/item/mauritius--approved-drug-list-for-public-hospitals-in-mauritius-2022-(english) [cited 2025 Oct 15].

[50] Mozambique: Lista Nacional de Medicamentos Essenciais 2017 (Portuguese). Maputo: Ministry of Health, Republic of Mozambique; 2017. Available from: https://www.who.int/publications/m/item/mozambique--lista-nacional-de-medicamentos-essenciais-2017-(portugues) [cited 2025 Oct 15].

[51] Namibia standard treatment guidelines, 1st ed. Windhoek: Ministry of Health and Social Services, Republic of Namibia; 2011. Available from: https://policyvault.africa/wp-content/uploads/policy/NAM27.pdf [cited 2025 Oct 15].

[52] Niger: Liste Nationale des Médicaments Essentiels 2018 (French). Niamey: Ministry of Public Health; 2018. Available from: https://www.who.int/publications/m/item/niger--liste-nationale-des-medicaments-essentiels-2018-(french) [cited 2025 Oct 15].

[53] Nigeria standard treatment guidelines, 2nd edition. Abuja: Federal Ministry of Health, Nigeria; 2016. Available from: https://www.medbox.org/document/nigeria-standard-treatment-guidelines [cited 2025 Oct 15].

[54] Rwanda standard treatment guidelines. Kigali: Ministry of Health, Republic of Rwanda; 2022. Available form: https://www.moh.gov.rw/index.php?eID=dumpFile&t=f&f=92519&token=bf359f5cf0f077357f3e69579f256ba9d0396939 [cited 2025 Oct 15].

[55] Sao Tome and Principe: lista nacional de medicamentos 2020 (Spanish). Sao Tome: Ministry of Health, Democratic Republic of Sao Tome and Principe; 2020. Available from: https://www.who.int/publications/m/item/sao-tome-and-principe--lista-nacional-de-medicamentos-2020-(spanish) [cited 2025 Oct 15].

[56] Senegal: liste nationale des médicaments et produits essentiels 2022 (French). Dakar: Senegalese Pharmaceutical Regulatory Agency, Ministry of Health and Social Action, Republic of Senegal; 2022. Available from: https://www.who.int/publications/m/item/senegal--liste-nationale-des-medicaments-et-produits-essentiels-du-senegal-2022-(french) [cited 2025 Oct 15].

[57] Standard treatment guidelines for Seychelles. Victoria: Ministry of Health, Seychelles; 2003. Available from: https://extranet.who.int/ncdccs/Data/SYC_D1_Standard%20Treatment%20Guidlines-Seychelles.pdf [cited 2025 Oct 15].

[58] Standard treatment guidelines 2021. Freetown: Ministry of Health and Sanitation, Government of Sierra Leone: 2021. Available from: https://mohs.gov.sl/download/71/pharmaceutical-service/18081/standard-treatment-guidelines_sierra-leone-2021-edition.pdf [cited 2025 Oct 15].

[59] Standard treatment guidelines and essential medicines list for South Africa. hospital level, adults, 2019 edition. Pretoria: The National Department of Health, South Africa; 2019. Available from: https://www.sapc.za.org/Media/Default/Documents/STG%20hospital%20level%20adult%202019_v2.0.pdf [cited 2025 Oct 15].

[60] South Sudan: essential medicines list (SSEML) 2018 (English). Juba: Ministry of Health, South Sudan; 2018. Available from: https://www.who.int/publications/m/item/south-sudan--essential-medicines-list-(sseml)-2018-(english) [cited 2025 Oct 15].

[61] Gambia: the Gambia standard drug treatment guide. Banjul: Department of State for Health & Social Welfare; 2001.

[62] Togo: liste nationale des médicaments essentiels sous dci pour les adultes 2021 (French). Lomé: Ministry of Health of the Togolese Republic; 2021. Available from: https://www.who.int/publications/m/item/togo--liste-nationale-des-m-dicaments-essentiels-sous-dci-pour-les-adultes-2021-(french) [cited 2025 Oct 15].

[63] Uganda clinical guidelines 2023: national guidelines for management of common health conditions. Kampala: Ministry of Health, Uganda; 2023. Available from: https://www.differentiatedservicedelivery.org/wp-content/uploads/UCG-2023-Publication-Final-PDF-Version-1.pdf [cited 2025 Oct 15].

[64] Standard treatment guidelines and national essential medicines list for Tanzania mainland, sixth edition. Dodoma: Ministry of Health, Community Development, Gender, Elderly and Children, United Republic of Tanzania; 2021. Available from: https://medicine.st-andrews.ac.uk/igh/wp-content/uploads/sites/44/2022/01/STG-NEMLIT-2021.pdf [cited 2025 Oct 15].

[65] Standard treatment guidelines, essential medicines list and essential laboratory supplies list for Zambia. Lusaka: Zambia National Formulary Committee, Ministry of Health; 2013.

[66] 8th essential medicines list and standard treatment guidelines for Zimbabwe. Harare: Ministry of Health & Child Care; 2020. Available from: https://www.differentiatedservicedelivery.org/wp-content/uploads/EDLIZ-2020-FINAL-for-circulation1-255.pdf [cited 2025 Oct 15].

[67] Zimbabwe National Healthy Ageing Strategic Plan 2017-2020. Harare, Geneva, London: Zimbabwe Ministry of Health and Child Care, World Health Organization, Age International; 2017. Available from: https://extranet.who.int/countryplanningcycles/sites/default/files/planning_cycle_repository/zimbabwe/zimbabwe_national_healthy_ageing_strategic_plan_2017-2020.pdf [cited 2025 Nov 13].

[68] Album D, Westin S. Do diseases have a prestige hierarchy? A survey among physicians and medical students. Soc Sci Med. 2008 Jan;66(1):182–8. 10.1016/j.socscimed.2007.07.003 17850944

[69] Fisher JM, Garside MJ, Brock P, Gibson V, Hunt K, Briggs S, et al. Why geriatric medicine? A survey of UK specialist trainees in geriatric medicine. Age Ageing. 2017 Jul 1;46(4):672–7. 10.1093/ageing/afx009 28164214

[70] Robbins TD, Crocker-Buque T, Forrester-Paton C, Cantlay A, Gladman JRF, Gordon AL. Geriatrics is rewarding but lacks earning potential and prestige: responses from the national medical student survey of attitudes to and perceptions of geriatric medicine. Age Ageing. 2011 May;40(3):405–8. 10.1093/ageing/afr034 21427112

[71] Goodman-Palmer D, Ferriolli E, Gordon AL, Greig C, Hirschhorn LR, Ogunyemi AO, et al. Health and wellbeing of older people in LMICs: a call for research-informed decision making. Lancet Glob Health. 2023 Feb;11(2):e191–2. 10.1016/S2214-109X(22)00546-0 36669801

[72] Global report on ageism. Geneva: World Health Organization; 2021. Available from: https://www.who.int/publications/i/item/9789240016866 [cited 2025 Apr 1].

[73] Delirium: prevention, diagnosis and management in hospital and long-term care. Clinical Guideline CG103 [internet]. London: National Institute for Health and Care Excellence; 2010. Available from: https://www.nice.org.uk/guidance/cg103/chapter/Recommendations#treating-delirium [cited 2025 Jun 17].

[74] Impact of US funding cuts on HIV programmes in East and Southern Africa [internet]. Geneva: UNAIDS; 2025. Available from: https://www.unaids.org/en/resources/presscentre/featurestories/2025/march/20250331_ESA-region_fs [cited 2025 Jun 17].

[75] Knights D, Knights F, Lawrie I. Upside down solutions: palliative care and COVID-19. BMJ Support Palliat Care. 2020 Jul 17;14 e1:e583–7. 10.1136/bmjspcare-2020-002385 32680888

[76] Parkinson disease: a public health approach. Technical brief. Geneva: World Health Organization; 2022. Available from: https://www.who.int/publications/i/item/9789240050983 [cited 2025 Oct 28].

[77] Kim DH, Rockwood K. Frailty in older adults. N Engl J Med. 2024 Aug 8;391(6):538–48. 10.1056/NEJMra2301292 39115063 PMC11634188

[78] O’Caoimh R, Sezgin D, O’Donovan MR, Molloy DW, Clegg A, Rockwood K, et al. Prevalence of frailty in 62 countries across the world: a systematic review and meta-analysis of population-level studies. Age Ageing. 2021 Jan 8;50(1):96–104. 10.1093/ageing/afaa219 33068107

[79] Oppong-Yeboah B, Amini N, van Uffelen J, Gielen E, Yawson AE, Tournoy J. Frailty and falls in community-dwelling older adults in sub-Saharan Africa: a scoping review. Arch Gerontol Geriatr Plus. 2024;1(4):100062. 10.1016/j.aggp.2024.100062

